# Middle Palaeolithic and Neolithic Occupations around Mundafan Palaeolake, Saudi Arabia: Implications for Climate Change and Human Dispersals

**DOI:** 10.1371/journal.pone.0069665

**Published:** 2013-07-24

**Authors:** Rémy Crassard, Michael D. Petraglia, Nick A. Drake, Paul Breeze, Bernard Gratuze, Abdullah Alsharekh, Mounir Arbach, Huw S. Groucutt, Lamya Khalidi, Nils Michelsen, Christian J. Robin, Jérémie Schiettecatte

**Affiliations:** 1 CNRS, UMR 5133 ‘Archéorient’, Maison de l'Orient et de la Méditerranée, Lyon, France; 2 School of Archaeology, Research Laboratory for Archaeology and the History of Art, University of Oxford, Oxford, United Kingdom; 3 Human Origins Program, Smithsonian Institution, Washington, D.C., United States of America; 4 Department of Geography, King’s College London, London, United Kingdom; 5 CNRS, UMR 5060 ‘Iramat’, Centre Ernest Babelon, Orléans, France; 6 Department of Archaeology, College of Tourism and Archaeology, King Saud University, Riyadh, Saudi Arabia; 7 Ministry of Higher Education, Riyadh, Saudi Arabia; 8 CNRS, UMR 8167 ‘Orient & Méditerranée - Mondes sémitiques’, Ivry-sur-Seine, France; 9 Technische Universität Darmstadt, Institut für Angewandte Geowissenschaften, Darmstadt, Germany; IPATIMUP (Institute of Molecular Pathology and Immunology of the University of Porto), Portugal

## Abstract

The Arabian Peninsula is a key region for understanding climate change and human occupation history in a marginal environment. The Mundafan palaeolake is situated in southern Saudi Arabia, in the Rub’ al-Khali (the ‘Empty Quarter’), the world’s largest sand desert. Here we report the first discoveries of Middle Palaeolithic and Neolithic archaeological sites in association with the palaeolake. We associate the human occupations with new geochronological data, and suggest the archaeological sites date to the wet periods of Marine Isotope Stage 5 and the Early Holocene. The archaeological sites indicate that humans repeatedly penetrated the ameliorated environments of the Rub’ al-Khali. The sites probably represent short-term occupations, with the Neolithic sites focused on hunting, as indicated by points and weaponry. Middle Palaeolithic assemblages at Mundafan support a lacustrine adaptive focus in Arabia. Provenancing of obsidian artifacts indicates that Neolithic groups at Mundafan had a wide wandering range, with transport of artifacts from distant sources.

## Introduction

The Arabian Peninsula is fast becoming a key region for understanding palaeoenvironmental change and its relationship to human occupation history. Major recent improvements have been made in our understanding of the Palaeolithic record of the region (see: [1], [2], [3]). Recent discoveries and investigations of stratified and dated archaeological sites have been focused on Middle Palaeolithic sites in Marine Isotope Stage (MIS) 5 and 3 (e.g. [4], [5], [6], [7], [8], [9], [10]). While these discoveries have done much to improve our understanding of the Arabian Middle Palaeolithic, large spatial and temporal gaps in our knowledge remain. Improvements have also been made in our understanding of the Neolithic sites dating to the Early and Middle Holocene, although much of this work has been centered on the extreme southern portions of the Peninsula and the Arabian Gulf region (e.g. [11], [12], [13], [14]).

**Figure 1:**
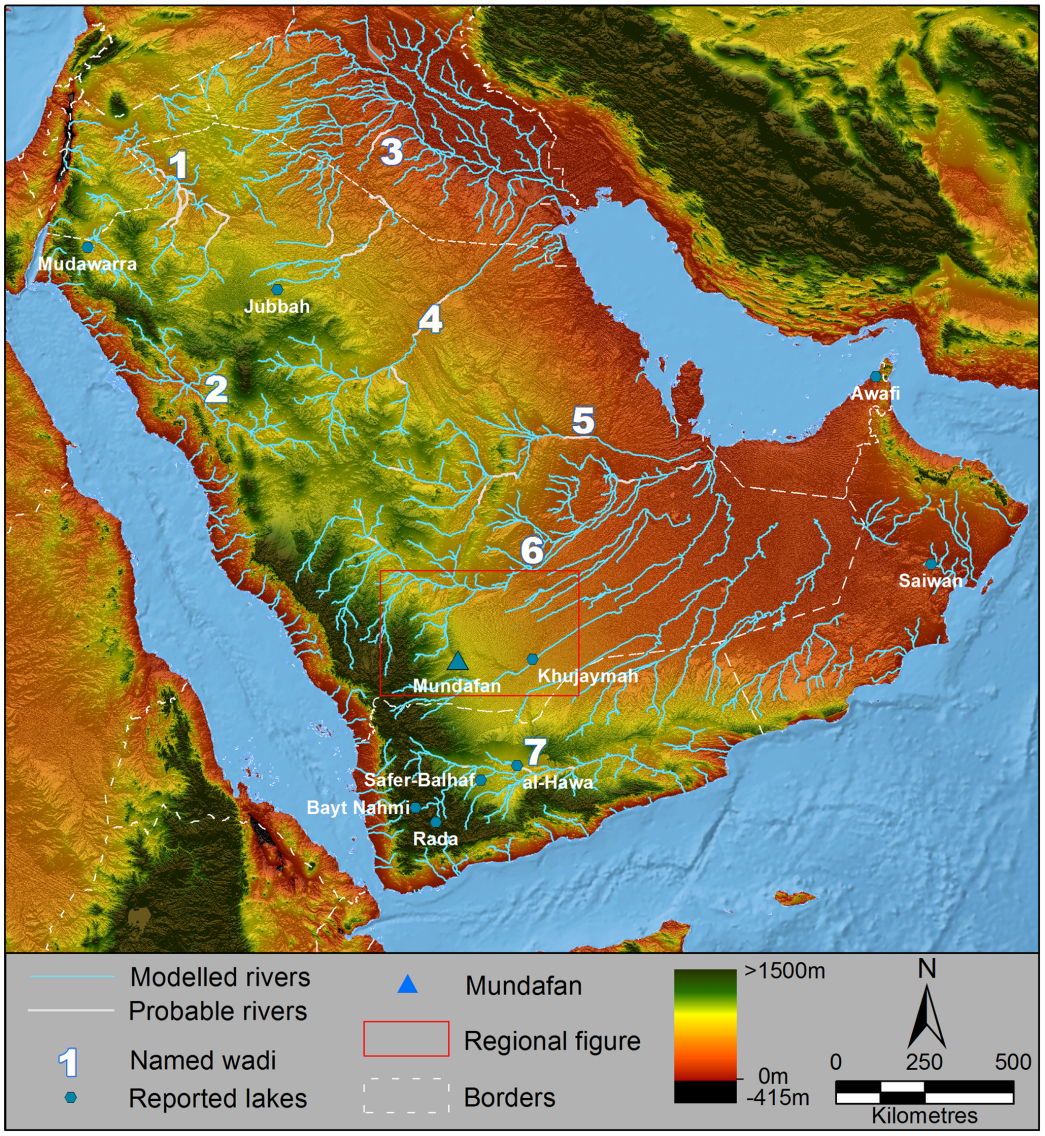
Palaeodrainage networks of Arabia. Key wadis are named, lakes discussed in the text located and labelled, and international borders area displayed by dashed lines. Drainage network data modeled through flow analyses (light blue) is superimposed upon SRTM V.4 elevation data [48], overlain upon Natural Earth 2 data for the oceanic regions. Interpreted channel connections potentially active during recent wet phases are marked in white. The red box outlines the region shown in figure 4. Major wadis are numbered: 1- Wadi as Sirhan, 2-Wadi al Hamd, 3- Euphrates and associated Widyan, 4-Wadi al Batin, 5-Wadi Sahba, 6- Wadi ad Dawasir, 7- Wadi Hadramawt.

Palaeoenvironmental studies of cave speleothems (e.g., [15], [16]) and palaeolakes (e.g. [17], [18], [19], [20], [21], [22], [23]) have provided insights about changing environments through time. Archaeological sites have been found associated with palaeolake shores, frequently identified on the basis of characteristic stone tool industries. The presence of the lakes in wet periods, together with the activation of major river systems [20], [24], [25], have been linked to hominin expansions [21], [22], [26], [27]. Both Late Pleistocene and Holocene relict lakes have been investigated in Mundafan and Khujaymah, Saudi Arabia [23], Mudawwara, southern Jordan [28], Jubbah, Saudi Arabia [21], [22], [29], [30], al-Hawa and Rada’, Yemen [18], [19], Safer-Balhaf, Yemen [31], Bayt Nahmi, Yemen [32], Saiwan, Oman [33], and Awafi, UAE [34], [35]. The majority of these studies have been dedicated to documenting environmental change, and less focus has been placed on investigating the relationships between the palaeolakes and human occupations. Figure 1 displays these lake locations, in addition to modeled palaeodrainage courses (see methods section).

Here we report the first reliable discoveries of Middle Palaeolithic and Neolithic sites in association with the former lakeshores of the Mundafan palaeolake, in the Rub’ al-Khali of Saudi Arabia. We associate our new archaeological finds with previous environmental research [36], [37] and recent geochronological and environmental reassessments, which demonstrates that the Mundafan palaeolake was an important focus for hunting during these two periods. Furthermore, these data establish that there was a *Homo sapiens* presence in the Arabian interior, which further supports the notion of a dispersal out of Africa [23], although no archaeological sites were reported before. We consider these sites in relation to the local and regional geomorphology and archaeology. The geomorphology is evaluated using GIS and remote sensing analyses of Landsat Thematic Mapper (TM) imagery and digital elevation models (DEM) while the regional archaeology is evaluated using a database developed by Groucutt and Petraglia [3].

## Methods

### Archaeological Methods

Given the importance of the environmental research carried out in the Mundafan palaeolake region, pilot archaeological research was undertaken. The present article reports on the discovery of both Middle Palaeolithic and Neolithic sites along the south-eastern shores of the palaeolake, suggesting that climatic cycles have most probably influenced the history of human occupations in Arabia. All necessary permits were obtained for the Najran-Mundafan fieldwork and analyses, which complied with all relevant regulations from the Saudi Commission for Tourism and Antiquities, Kingdom of Saudi Arabia.

**Figure 2:**
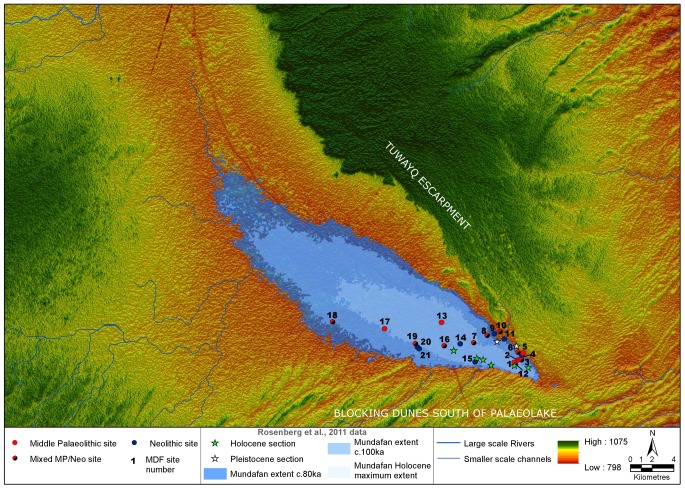
Newly discovered archaeological sites (by period) overlain upon the palaeohydrological reconstruction of the Mundafan area. With palaeolake section locations and inferred extent data from Rosenberg et al., [23]. Data is overlain upon Aster GDEM2 elevation data.

**Figure 3:**
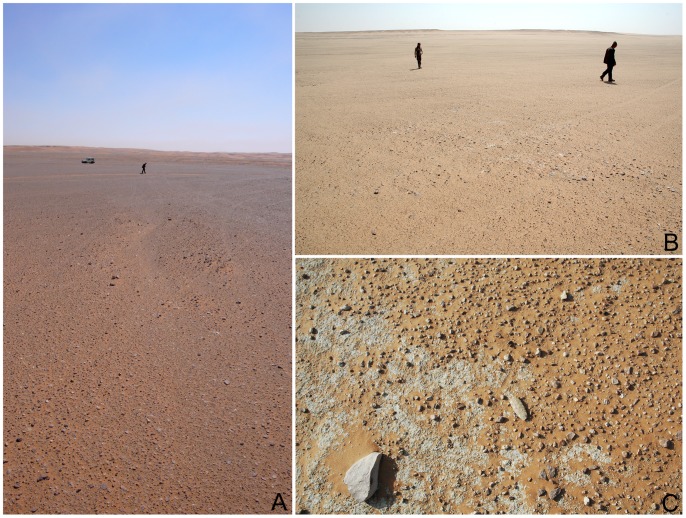
Views of surface lithic scatters at Mundafan. A: MDF-01 site; B: MDF-20 site; C: a fragmentary bifacial piece on the surface of MDF-12 site (length of the artifact: 91 mm).

The two brief reconnaissance surveys at Mundafan took place in 2010 and 2011, along the southern and south-eastern lakeshores. Several spots in the central part of the palaeolake were also visited in order to determine the presence or the absence of archaeological sites. A total of 21 lithic surface scatters (sites) were discovered, labeled MDF-01 to MDF-21 (Fig. 2). All of the sites are located within the palaeolake basin, with many showing an association with suggested palaeolake shorelines, with a generally low to moderate density of archaeological material (<1 to 1–5 artifacts per m^2^). The lithic scatters are exclusively characterized by the presence of flakes, cores and tools, and no other kinds of archaeological remains were identified. The temporal delimitation of the sites is sometimes difficult to precisely determine, as their status of proper individual ‘site’ can be biased by repeated occupations (Fig. 3). Lithic artifacts were systematically collected from sites (n = 1009, Table 1), though when dense, a selective sample of only diagnostic pieces (tools, cores, blades and technologically informative pieces such as debordant flakes) was made. The raw material used in stone tool knapping is typically a fine grained chert of relatively good quality, the source of which is not yet known. Nearby limestone cliffs of the Tuwayq Mountains, a few hundred meters to the northeast, may have provided a primary source. Chert gravels are present on the surface of the lakeshores, but are too small to be used as knappable chert blocks. Much rarer raw materials were observed: allochtonous obsidians, ferruginous quartzite, and small nodules of poor quality vein quartz. Workable fossil wood nodules were also noticed, but only as natural unworked pieces. Two main periods are recognized by lithic typology: (1) Middle Palaeolithic, and (2) aceramic Neolithic of the Rub’ al-Khali tradition.

**Table 1:** Lithic artifacts discoveries from Mundafan palaeolake, selective collection from surface sites.

SITE	flakes	cores forflakes	blades	retouchedflakes & tools	Levalloiscores	Levalloisflakes	debordantflakes	TOTAL
MDF-01	203	1	14	18	9	15	4	**264**
MDF-02	8			1		1		**10**
MDF-03	15			6				**21**
MDF-04	17	1		2				**20**
MDF-05	17			13		1		**31**
MDF-06	4	1		3				**8**
MDF-07	23	1						**24**
MDF-08	23			1				**24**
MDF-09				6				**6**
MDF-10	7				1			**8**
MDF-11	20	1		5				**26**
MDF-12				17				**17**
MDF-13	4							**4**
MDF-14		1						**1**
MDF-15	169			6				**175**
MDF-16	16			7		4		**27**
MDF-17	39				1	5		**45**
MDF-18	5							**5**
MDF-19	21							**21**
MDF-20	140	4	9	62				**215**
MDF-21	22			35				**57**
**TOTAL**	**753**	**10**	**23**	**182**	**11**	**26**	**4**	**1009**

**Figure 4:**
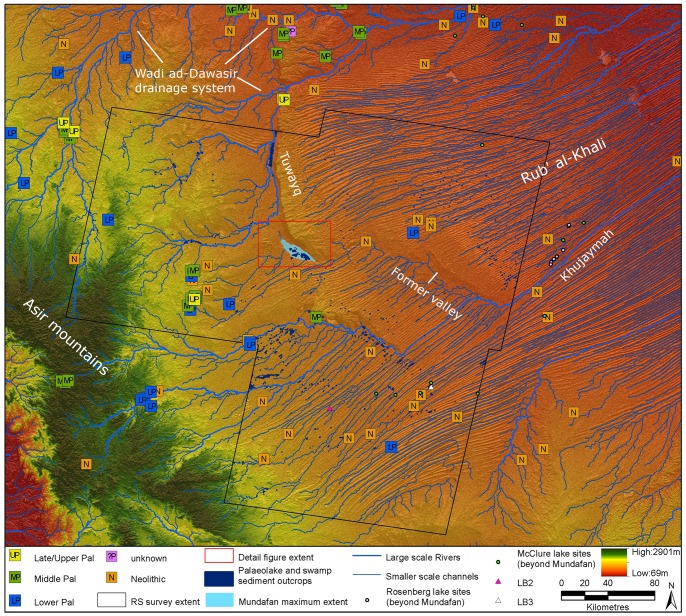
Regional archaeology and palaeohydrology of the Mundafan area. The remote sensing (RS) survey area is the region within which we have used the MF/SAM method to map palaeolake sediment and swamp outcrops (results displayed). Archaeological sites (by period, data from [3]), modeled drainage, the maximum recorded extent of Mundafan palaeolake [23], LB2 and LB3 site locations and published palaeolake sediment occurrences [23], [37] are displayed. All data is overlain upon SRTM V.4 elevation data [48].

The distribution of archaeological sites in Figure 4 shows the approximate positions of archaeological localities identified by the ‘Comprehensive Archaeological Survey Programme’ of the Kingdom in the 1970’s. Locations were calculated from the maps produced by this survey by measuring from the grid lines on the original publications. Wherever possible, efforts were made to correlate features shown on the original maps with satellite imagery in order to check the accuracy of the calculated locations. The information was compiled into a database which was used to generate maps of site distribution in Groucutt and Petraglia, 2012 [3]. The attribution to cultural phases (e.g. Lower Palaeolithic, Neolithic, etc.) reflects the terminology of the discoverers, and should be interpreted with caution. In particular ‘Upper Palaeolithic’ is a problematic designation in Arabia (e.g. [38]). The problems of the original survey methodology aside, Figure 4 reveals the widespread evidence for prehistoric occupation in the western Rub’ al-Khali area. While we currently have a poor grasp on chronological and techno-typological variability, it is clear that the area has been repeatedly inhabited by hominins. In addition there appears to be a correlation of archaeological localities with palaeorivers. The correlation of prehistoric archaeological evidence with palaeorivers and palaeolakes seems to be a common feature in Arabia.

### Palaeohydrological Mapping

In order to further our understanding of the palaeohydrology of the Lake Mundafan Basin and the surrounding regions, and the relationship of the palaeoriver network to the other large palaeoriver systems of the Arabian peninsula, DEMs and Landsat TM imagery was analyzed to map channel networks and palaeolake sediment outcrops. It should be noted that DEM data relate to the modern landscape, and that Pleistocene palaeohydrology may have been different, prior to episodes of drainage capture or dune emplacement. For this reason, DEMs and flow data were examined in concert with satellite imagery and palaeolake remote sensing analyses to allow the interpretation of past landscape change. Regional palaeochannel networks were derived using GIS flow analyses [39] based upon the Hydrosheds 3 arc-second resolution global hydrology dataset [40], following a method formerly utilized in the Nefud region in northern Saudi Arabia [22]. Networks were derived at a range of flow accumulation thresholds (c. 1000km^2^, 100km^2^ and 50km^2^ contributing area at the equator) to allow clear examination of the palaeohydrological characteristics of the region at a range of scales (Fig. 1, 2 and 4).

Regional palaeolake deposits were identified through analysis of three cloud-free Landsat TM scenes covering the region to the west of Mundafan, the Mundafan area itself, and the western portion of the Rub’ al-Khali to the east and southeast (Fig. 4). Atmospheric correction and conversion to top-of-atmosphere reflectance were applied to the optical bands of the Landsat scenes (Bands 1–5 and band 7). Evaporite deposits (predominantly Gypsum) associated with terminal desiccation phases of palaeolake events have been documented within the Mundafan basin [37], and large areas of these deposits can be clearly discerned using band 7,4,1 RGB false color composite (FCC) images that have been shown to be effective for identifying comparable palaeolake sediments in the Sahara [41]. To facilitate the automated detection of comparable deposits within the wider region, training sites were selected from within the Mundafan basin deposits and other regional evaporites and then Spectral Angle Mapper classification [42] and Matched Filtering [43] procedures were applied to the Landsat TM imagery. The results of these two analyses were used in a band ratio (MF/SAM), reducing false positives and accentuating materials with the greatest spectral similarity to the input training sites. A range of thresholds were iteratively applied to this ratio image to establish an optimum threshold between lake sediments and other spectrally similar materials such as limestone bedrock. The chosen threshold shows the greatest conformity to the extent of the known deposits within the Mundafan basin while minimizing overestimation. Finally a majority filter (8×8 cells) was applied to minimize single-cell misclassifications.

Results from this filtered optimum threshold data were examined in comparison to the 7,4,1 FCC images, and some mapped areas were clearly not associated with former palaeolakes, these were deposits with similar spectral properties, primarily limestones from the crest of the Tuwayq escarpment and outwash surfaces to the west of Mundafan. These false positives errors were masked out. The final data (Fig. 4) shows a high degree of correspondence with the FCC data, identifying clear palaeolake and fluvial swamp materials west of Mundafan, and interdune responses in the western Rub’ al-Khali likely to represent interdune lake deposits, a phenomenon well documented in the region [23], [36], [37].

### Radiocarbon Dating

While no remains of ostrich have yet been identified in the Mundafan region, ostrich eggshells are common. For radiocarbon dating selected pieces were treated with 0.5M HCl to remove the possibly contaminated outer layers. The remaining material was reacted with concentrated H_3_PO_4_ in a vacuum line and the generated CO_2_ was reduced with H_2_ and Fe powder acting as a catalyst to graphite following the procedures described in Czernik and Goslar [44]. The resultant graphite was analyzed for 14C by Accelerator Mass Spectrometry (AMS; [45]). After correction for isotopic fractionation based on the simultaneously measured 13C/12C ratio, radiocarbon ages were calculated. Calibration was performed using the software OxCal 4.1 [46] and the calibration curve IntCal09 [47]. While the roots casts date to the early Holocene, the ostrich egg shells show ages close to or beyond the ^14^C dating limit (Table 2).

**Table 2:** Published palaeolake dates from Mundafan and the western Rub’ al-Khali, after McClure’s studies [36], [37], and those of Rosenberg et al., [23].

Source	Location	Sample	Methods	Type	Period	Date (Ka)	Error (1σ Ka)	Notes
**Mundafan basin**								
[**36**] **, ** [**37**]	1-23	Uga-1216	14C	Algal	H	6.1	0.07	–
[**36**] **, ** [**37**]	1-3-2	Uga-1207	14C	Marl	H	7.04	0.115	–
[**36**] **, ** [**37**]	1-2-2	Uga-1204	14C	Marl	H	7.19	0.085	–
[**36**] **, ** [**37**]	1-3-4	Uga-1206	14C	Marl	H	7.265	0.08	–
[**36**] **, ** [**37**]	1-3-8	Uga-1208	14C	Shell	H	7.4	0.21	–
[**36**] **, ** [**37**]	1-3-6	Uga-1205	14C	Marl	H	7.77	0.09	–
[**37**]	1-19-3	Beta-5111	14C	Shell	H	7.84	0.14	+
[**23**]	22.3	12	14C	Organics from marl	H	7.86	0.1	corrected date consistent with OSL
[**37**]	1-3-8	Beta-5103	14C	Shell	H	8.05	0.13	+
[**36**] **, ** [**37**]	1-7-1/2	Uga-1212	14C	Marl	H	8.06	0.095	–
[**36**] **, ** [**37**]	1-26-1	Uga-1221	14C	Marl	H	8.155	0.085	–
[**23**]	23.2	10	14C	Organics from marl	H	8.16	0.15	upper marl
[**37**]	1-26	Beta-5113	14C	Shell	H	8.31	0.15	+
[**36**] **, ** [**37**]	1-26-1	Uga-1222	14C	Shell	H	8.565	0.11	–
[**36**] **, ** [**37**]	1	Uga-1214	14C	Ashy Marl	H	8.8	0.09	–
[**23**]	23.1	1	OSL	Sand below marl	H	8.8	0.4	
[**23**]	21.2 (A)	29	14C	Organics from marl	H	9.22	0.19	central corrected date (dates range from 10.2ka–8.2ka)
[**23**]	21.1	16	14C	Organics from marl	H	9.29	0.15	corrected date consistent with OSL
[**37**]	1-20-4	Beta-5102	14C	Shell	H	9.36	0.13	+
[**23**]	22.6	7	OSL	Sand below marl	H	9.8	0.6	upper marl
[**36**] **, ** [**37**]	1-22-1	Uga-1215	14C	Marly Siltstone	H	11.465	0.115	**–**
[**23**]	23.3	4	OSL	Sand below marl	H	12	0.7	
[**23**]	23.2	9	OSL	Sand below marl	H	14.4	1	lower marl
[**37**]	1-27-2	GX-4199	14C	Marl	P	14.73	0.29	–
[**36**] **, ** [**37**]	1-6	Uga-1210	14C	Marl	P	14.965	0.195	–
[***36***] ***, *** [***37***]	*1-18-1*	*Uga-1218*	*14C*	*Marl*	*P*	*17.46*	*0.245*	–
[**23**]	22.6	6	OSL	Sand below marl	H	19.1	1.6	lower marl
[***37***]	*1-11-3*	*Beta-5112*	*14C*	*Shell*	*P*	*20.69*	*0.44*	+
[***36***] ***, *** [***37***]	*1-1-2*	*Uga-1203*	*14C*	*Marl*	*P*	*21.09*	*0.42*	–
[***36***] ***, *** [***37***]	*1-9*	*Uga-1217*	*14C*	*Marl*	*P*	*21.28*	*0.275*	–
[***36***] ***, *** [***37***]	*1-1-1*	*Uga-1202*	*14C*	*Marl*	*P*	*22.345*	*0.415*	–
[***37***]	*1-5*	*Uga-1211*	*14C*	*Marl*	*P*	*22.965*	*0.39*	–
[***36***] ***, *** [***37***]	*1-4*	*Uga-1209*	*14C*	*Marl*	*P*	*23.075*	*0.425*	–
[***36***] ***, *** [***37***]	*1-8*	*Uga-1213*	*14C*	*Marl*	*P*	*24.125*	*0.4*	–
[***36***] ***, *** [***37***]	*1*	*I-7427*	*14C*	*Oolite*	*P*	*25.66*	*0.81*	–
[***36***] ***, *** [***37***]	*1-25-1*	*Uga-1220*	*14C*	*Marl*	*P*	*28.75*	*0.615*	–
[***36***] ***, *** [***37***]	*1-18-1*	*Uga-1219*	*14C*	*Shell*	*P*	*29.595*	*0.78*	–
[***37***]	*1-24*	*GX-4197*	*14C*	*Kunkar*	*P*	*30.11*	*1.95*	–
[***37***]	*1-18*	*Beta-5107*	*14C*	*Shell*	*P*	*32.14*	*0.85*	–
[***36***] ***, *** [***37***]	*1*	*I-7111*	*14C*	*Shell*	*P*	*36.3*	*2.4*	–
[**23**]	22.5	48	OSL	Sand below marl	P	79	7	
[**23**]	22.2	38&46 Weighted Mean	OSL	Sand	P	101.72	4.8	our average of bracketing dates
**Rub’ al-Khali palaeolakes**								
[**37**]	16- 2	Beta-5108	14C	shell	H	6.27	0.1	+
[**37**]	Wadi Dawasir	GX4192	14C	Marl	H	6.61	0.15	–
[**37**]	2	Uga-1419	14C	tubule scree	H	6.885	0.075	–
[**36**] **, ** [**37**]	Nadqan	I7307	14C	shell	H	7.16	0.115	–
[**37**]	3-2	Uga-1418	14C	shell	H	7.21	0.09	–
[**37**]	North RAK	Uga-1748	14C	shell	H	7.395	0.14	**–**
[**37**]	18	GX4194	14C	shell	H	7.66	0.21	–
[**37**]	18-7	Beta-5106	14C	shell	H	7.78	0.9	+
[**37**]	18	GX4195	14C	shell	H	7.885	0.19	**–**
**This study**	LB2	49820	14C	Root tube	H	9.14	0.07	Neolithic lithics
[**37**]	SW RAK	Uga- 1415	14C	tubule scree	H	8.215	0.125	**–**
**This study**	LB2	49819	14C	Root tube	H	10.50	0.08	Neolithic lithics
[**37**]	Wadi Dawasir	GX4193	14C	marly siltstone	H	9.475	0.275	–
[**37**]	5-3	Uga-1416	14C	marly claystone	H	9.605	0.125	–
[**37**]	6-3	Uga-1417	14C	Marl	P	12.315	0.12	–
[***37***]	*11-2*	*Beta-5114*	*14C*	*chalk*	*P*	*17.51*	*0.31*	+
[***37***]	*12-7/2*	*GX4191*	*14C*	*Marl*	*P*	*20.845*	*0.575*	–
[***37***]	*15-4*	*I-6987*	*14C*	*Marl*	*P*	*21.4*	*0.45*	–
[***37***]	*Central RAK*	*Beta-5110*	*14C*	*shell*	*P*	*23.82*	*0.59*	+
[***37***]	*SW RAK*	*GX4198*	*14C*	*Marl*	*P*	*24.18*	*0.765*	**–**
[***37***]	*Central RAK*	*Beta-5104*	*14C*	*shell*	*P*	*26.91*	*0.45*	+
[***37***]	*19-3*	*Uga-1747*	*14C*	*Marl*	*P*	*27.13*	*0.555*	–
[***36***] ***, *** [***37***]	*19-2*	*I-7447*	*14C*	*chalk*	*P*	*27.16*	*0.94*	–
[***37***]	*SW RAK*	*GX4196*	*14C*	*Marl*	*P*	*27.495*	*1.05*	–
***This study***	*LB2*	*49818*	*14C*	*Ostrich egg shell*	*P*	*49.8*	*3.1*	*near 14C dating limit*
***This study***	*LB3*	*49822*	*14C*	*Ostrich egg shell*	*P*	*>50*	*–*	*beyond 14C dating limit*
[**23**]	28.6 (B)	54	OSL	Sand below marl	P	88	6	
[**23**]	28.4	58	OSL	Sand below marl	P	90	9	
[**23**]	28.5	63	OSL	Sand below marl	P	113	10	
[**23**]	28.1	61	OSL	Sand below marl	P	121	7	
[**23**]	26.3 (D)	69	OSL	Sand below marl	P	136	14	upper marl
[**23**]	26.3 (D)	65	OSL	Sand below marl	P	120	10	lower marl
[**23**]	26.6	78	OSL	Sand below limestone	P	143	11	
[**23**]	25.3	75	OSL	Sand below marl	P	144	9	
[**23**]	25.4	74	OSL	limestone	P	147	15	

Dates in italics are suggested to be potentially older than reflected by their radiocarbon dates, in light of the results of the Rosenberg et al., [23] study. Radiocarbon dates from the McClure studies indicated with a+symbol were δ13C corrected, while a - symbol denotes uncorrected dates.

## The Pleistocene and Holocene of Palaeolake Mundafan and Surrounding Regions

**Figure 5:**
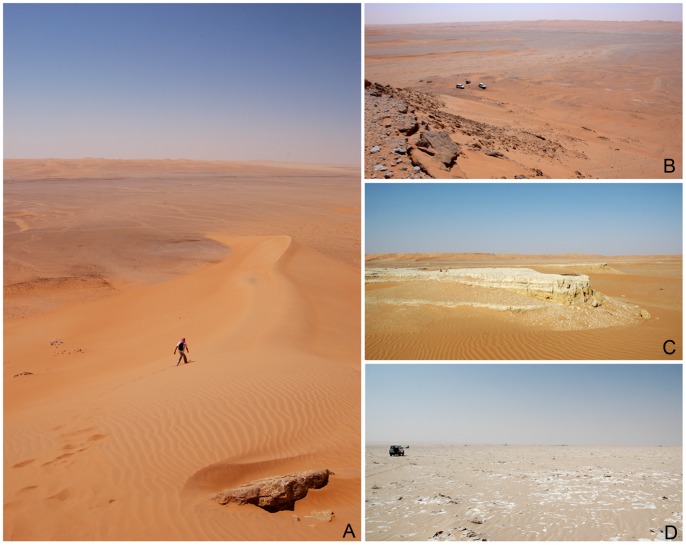
General views of the Mundafan palaeolake. A: from Jebel Tuwayq to the West; B: from Jebel Tuwayq to the South; C: at MDF-12, remnants of lacustrine deposits, Jebel Tuwayq is in the background; D: in the middle of the Holocene palaeolake with its typical whitish indurated crust of gray marls.

The Mundafan basin (18°34’N, 45°19’E), located in the southern Saudi Arabian province of Najran, represents one of the largest lacustrine deposits on the Arabian Peninsula, formerly radiocarbon dated by Mcclure to MIS 3 [37]. Recent research refutes this date, with Rosenberg et al., [23] using OSL dating to show that it formed during MIS 5 and had a surface area of up to ∼300 km^2^ (Fig. 2). This discrepancy in ages is now recognized as a common problem, and is associated with contamination of older radiocarbon dates during subsequent wet phases [23]. The bottom of the Lake Mundafan depression is relatively flat and only fluctuates between 860 and 870 m above sea level (asl). The lacustrine sediments are visible as an indurated crust of gray marls, deposited on dune sands (Fig. 5). Examining the reported heights of these lake sediments in relation to the SRTM DEM [48] we estimate an extent of about 58 km2 during the Holocene, 210 km^2^ at 80 ka and 100 km^2^ at 100 ka (Fig. 2). These are minimum estimates as the lake was almost certainly larger than the maximum height of the preserved sediments given the significant amount of deflation that must have occurred since the above mentioned lacustrine phases ceased. We estimate the maximum size the lake can form in the basin before it overflows into the river system to the north is 346 km^2^. Thus the lake was large at various times in the past, but its actual size cannot be determined exactly and varied between different wet phases, being particularly small during the Holocene. Our analyses utilize the results from the recent geochronological investigations at Mundafan by Rosenberg et al., [23] which provide the best current chronologies available for the basin, however substantial further work will be required in order to conclusively define the morphology of the palaeolake during the different humid phases of the late Pleistocene.

The lake is found at the juncture between the Asir mountains, the Tuwayq mountains and the Rub’ al-Khali desert (Fig. 4). The valley that contains the lake is delimited to the east by the southern extremity of the Tuwayq Mountains, the longest and highest of a series of Jurassic limestone escarpments in central Saudi Arabia that end about 60 kilometers south of Mundafan, after running along a 1300 km distance from the Nefud Desert in northern Arabia (Fig. 1, 2 and 4). To the west are the foothills of the Asir Mountains and to the south the Mundafan basin is surrounded by sand dunes that are part of the southwest margin of the Rub’ al-Khali desert.

The palaeohydrological reconstruction of the region shows that the principal source of water for the lake is via channels draining the Asir Mountains (Fig. 4). The neighboring cliffs of the Tuwayq mountains reach ca. 900–1000 m asl, but the vast majority of the drainage is to the east, away from Lake Mundafan and into the Rub’ al-Khali. The Tuwayq mountains gradually decline in altitude towards the south, briefly disappearing under the dunes of the Rub’ al-Khali (Fig. 2 and 4). If we look at the distribution of the sand dunes in the vicinity of Lake Mundafan in relation to the rest of the topography then it appears that prior to the development of the dunes the channels that drain into Lake Mundafan from the Asir Mountains would have flowed through the gap in the Tuwayq escarpment at the south-eastern end of the Mundafan Basin (Fig. 2 and 4) and along the broad north-west to south-east trending valley now underlying the dunes of the Rub’ al-Khali. This valley appears to have been carved by an ancient river system that was developed long before the emplacement of the Rub’ al-Khali dunes and is now partly obscured and blocked by them (Fig. 4). The blocking of the channel by dunes that were funneled through the gap in the escarpment led to the formation of the closed basin within which Lake Mundafan developed during subsequent humid periods (Fig. 2).

This ancient drainage course is visible as a valley partially exposed beneath obscuring dunes in satellite imagery and DEMs, and is corroborated by the flow analyses, which plot a drainage course following this system, seen as the large scale rivers presented in figures 1 and 4. The existence of such a system in the Rub’ al-Khali has previously been postulated by a number of authors (e.g. [49], [50], [51], [52], [53]). Our analysis shows that this large-scale river system is associated with the course of Wadi Najran and other wadis emanating from the Asir Mountains that are now, in places, partly buried by dunes. This drainage system occurs alongside a more dense, and perhaps more recent, network, both within the Rub’ al-Khali and emanating from the surrounding mountains (Fig. 4). The palaeolake sediment mapping reveals 3100 individual lake sediment exposures within the area of study. If we group those outcrops that appear to be related to a single large deposit, then we have approximately 1900 distinct palaeolake outcrops (Fig. 4). Thus there is much evidence for past humidity in the region, though some of this evidence is stronger than others. For example the river channels mapped in the mountains can be seen in the Landsat TM imagery, thus verifying their existence. However, in the Rub’ al-Khali we have mapped a very high channel density, yet inspection of the Landsat TM imagery shows no clearly defined channels, presumably because of sand movement in the recent arid phase, but also possibly because we have overestimated the channel density in the region. Notwithstanding this the results suggest regional humidity at times during the past.

Different views have been published regarding the activity of these drainage channels within the Rub’ al-Khali, with the most recent activity being ascribed to pluvial phases during the early Pleistocene [50], undifferentiated Pleistocene [51], and later Pleistocene [52], [53]. McClure [49] suggested that during Pleistocene pluvial phases increased surface sheet flooding was the dominant hydrologic factor in the dune regions, with no wadi through-flow. In contrast, Atkinson et al. [54] show that perennial fluvial activity occurred on the eastern margins of the Rub’ al-Khali during MIS 5e, 5a and the early Holocene, with a major dune building phase in late MIS 3, thus demonstrating that perennial rivers developed in the eastern part of the sand sea. Therefore, whether these topographic lows and interdune depressions, that are responsible for smaller scale channels plotted by the flow model in the Rub’ al-Khali (Fig. 4), would indeed be capable of carrying perennial flow during pluvial phases when flow was in excess of evaporative and infiltration processes remains unclear, but evidence is now becoming available that suggests they would [54].

McClure [37], [49] recognised numerous Pleistocene palaeolakes in the Rub’ al-Khali, many of which preserve freshwater mollusks suggesting perennial lake conditions in the region. One of these lakes at Khujaymah (Fig. 1 and 4) has been dated by Rosenberg et al., [23] to ca. 125 ka, indicating humid conditions in MIS 5e. A later pluvial period is indicated by palaeolake deposits emplaced in interdune depressions during the Holocene (mainly ca. 6–9 ka; see Table 2; [36], [37]). However, these Holocene palaeolakes have been interpreted as being often short-lived, of playa form, and derived solely from localized dune run-off [37], [49]. Nevertheless, this interpretation contrasts somewhat with the large number of palaeolakes we have identified from the remote sensing imagery, with the reported freshwater and grassland faunal assemblages associated with some of the Holocene deposits [37], [49], and with the broad regional distribution of Neolithic archaeological localities throughout much of the Rub’ al-Khali (Fig. 4) [3]. All these factors imply a sustained availability of local fresh water. It is clear however that some of the Rub’ al-Khali palaeolakes were of an ephemeral nature. For example we have identified interdune lacustrine silt and sand deposits (Fig. 4, LB2 and LB3) that lack freshwater mollusks but are fringed by calcareous root casts that date to the early Holocene (Table 2) and ostrich egg shell fragments, one of which dates to 49.8 ka with the other providing an infinite age (Table 2). Given the problems associated with older radiocarbon dates in the region [23] (see below for a more detailed discussion), these dates probably represent a wet phase prior to 50 ka (Table 2).

### Lake Mundafan

The first serious investigations of the Mundafan palaeolake occurred in the late 1970’s and early 1980’s, thanks to the pioneering efforts of Harold McClure [36]. A stratigraphic sequence more than 20 m in depth was reported and the results of 56 radiocarbon ages documented two main episodes of sedimentary infilling, the most ancient phase was estimated to date to 36 to 17 ka, and the more recent, to between 10.5 to 6 ka (Table 2) [36], [37], [55]. Fossilized Pleistocene and Holocene faunal remains were discovered in the lacustrine sediments of the Mundafan and nearby lake deposits in the southwest Rub’ al-Khali, revealing large mammal species such as wild goat and sheep (*Capra* sp./*Ovis* sp.), oryx (*Oryx* sp.), gazelle (*Gazella* sp.), horse and ass (*Equus* sp.), camel (*Camelus* sp.), wild cattle (*Bos primigenius, Bubalus* sp.), *Hippopotamus amphibious*, and birds such as ostrich *Struthio* sp. ([37] p.179). Most of these species are ungulates living in grassland and open woodlands, with large foraging ranges. However, the presence of Hippopotamus suggests that Palaeolake Mundafan was at times a deep permanent water body, presumably fed by perennial rivers from the Asir Mountains. Thus the local environment might well have consisted of riparian forests close to rivers and lakes with open savannah in less well watered areas.

Recent environmental and geochronological study at Mundafan confirms the Upper Pleistocene and Early Holocene lake formations [23], but does not support a humid period between 36 and 17 ka [36], [55]. The hypothesis of a late MIS 3 wet phase probably reflects problems with the use of radiocarbon method on bulk samples (see e.g. [20]). Optically Stimulated Luminescence (OSL) ages, faunal and floral remains, as well as sedimentological evidence from two sections that were located near our archaeological discoveries (Fig. 2), indicate wet phases in MIS 5c (ca. 100 ka) and MIS 5a (80 ka) (Table 2) [23]. Rosenberg et al. also investigated palaeolakes at Khujaymah in the inter-dune depressions of the Rub’ al-Khali [23]. These lake sediments also suggested a freshwater lake, but dating to ca. 125 ka. These results indicate three principal humid periods during MIS 5 that permitted the development of savannah, populated by herbivores. Different water levels have been observed in sections, showing intervals when the lake was slightly brackish due to high evaporation. The lack of evidence for humidity in MIS 5d (ca. 115–105 ka) and MIS 5b (95–85 ka) might suggest a return to arid conditions and a hostile desert-like landscape, as indicated by speleothem records in Oman and Yemen [56], [57], and by dune movements in Oman [58], [59]. Interestingly the dunes at the southern end of Lake Mundafan that have formed the closed basin in which the palaeolake developed during humid periods must be older than the oldest palaeolake sediments found in the basin (MIS 5), making them MIS 6 at the latest. Such an age is consistent with the oldest aeolian sediments found in the Rub’ al-Khali by Preusser [15].

There is now considerable evidence in Arabia that periods of humidity and aridity have alternated throughout the Middle and Upper Pleistocene with humid phases of varying intensity during MIS 9, 7, 6, 5 and 3 [20], [60], and some limited evidence for regional humidity during MIS 10 and 11 [61], [62], [63]. These humid periods reflect high summer insolation, pulling the Intertropical Convergence Zone (ITCZ) into southern Arabia, with accompanying summer monsoonal precipitation activating the river systems and filling the perennial lakes in the interdune depressions and enclosed basins in the southwest Rub’ al-Khali ([16], [23], [33], [36], [37], [49], [64]) and faunal associations commonly implying freshwater conditions at lake maxima, and a regional savannah grassland setting [37], [49]. Spatially extensive Pleistocene palaeolake events in the Mundafan basin contrasted with smaller-scale ‘shoe-string’ lake formations generated along the shallow interdune depressions [37], [49].

A later lacustrine interval occurred at Mundafan during the Early Holocene moist phase in southern Arabia, as evidenced by (Table 2). Freshwater mollusks attest to low salinity in the Early Holocene [37], [65] while the ostracode species assemblage confirms shallow-water environments with freshwater conditions in a perennial lake, and plant remains attest a vegetated lake fringed by reeds and C3 plants in a wetter and cooler environment than today. However, the occurrence of more saline intervals is attested to by the presence of benthic foraminifera species *Helenina anderseni* and *Trichohyalus aguayoi* that are characteristic of mangrove swamps, salt marshes and lagoons and were most probably brought to Mundafan by wading birds [66]. The only vertebrate remains found in section by Rosenberg et al. [23] are of gazelle, dated to 8.7 ka. These findings of early Holocene humidity are supported by regional climate records [15], [17], [19], [36], [67], [68], [69]. Speleothems from Oman provide a record of pluvial intervals between 10.5–6 ka BP [56], [57], [69], [70] when the monsoon was of sufficient power to reach the whole south of the Arabian Peninsula, thereby influencing the expansion of favorable habitats where human communities could develop Neolithic economies [14]. The Mundafan lake was nonetheless much reduced in extent at this time, with an area of ∼50 km^2^ and a maximum depth of 10 m (Fig. 2).

## Archaeological Investigations

Archaeological investigations in the Mundafan basin have been limited, and the region has been sparsely explored owing to its inhospitable environment [36], [71], [72]. In the first initial surveys in the 1970s and 1980s, Neolithic tool assemblages were reported, but briefly described, and Palaeolithic artifacts were never mentioned. The limited archaeological research that had occurred was not integrated with McClure’s palaeoenvironmental studies at Mundafan. McClure, however, investigated the Bani Khatmah site, ∼30 km from the lake basin, identifying ‘Aterian’ points, and connecting the lithic industry to the lake’s occupation in the early phase [73]. The absence of direct dating at the site, and the typologically indistinct nature of the tanged implements, has, however created doubts about cultural affiliation with the Aterian [2], [74].

### Middle Palaeolithic

**Figure 6:**
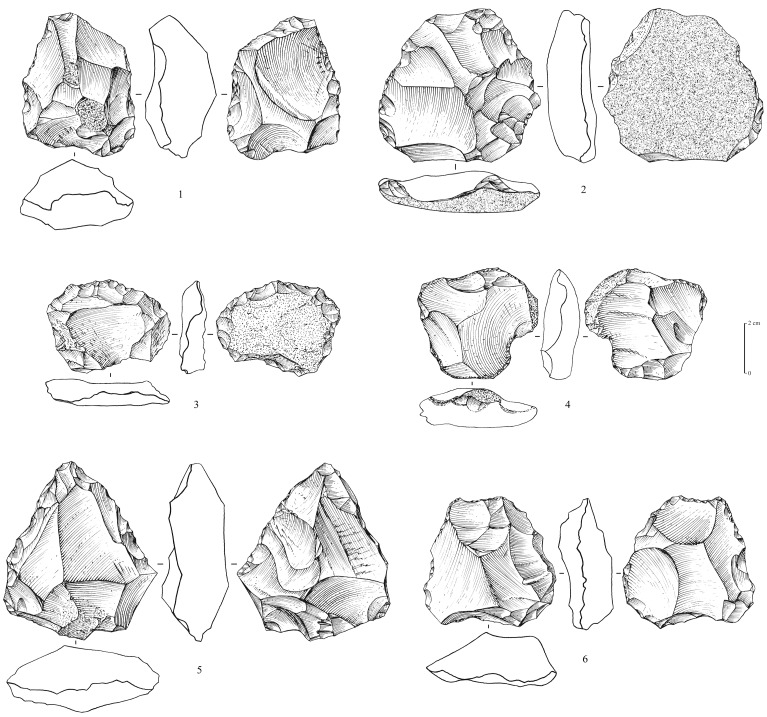
Levallois cores from Mundafan, in chert. 1,2,5,6: recurrent centripetal Levallois cores (5 might have been reused as a tool); 3,4: preferential Levallois cores with centripetal preparation. Drawings by G. Devilder, CNRS.

**Figure 7:**
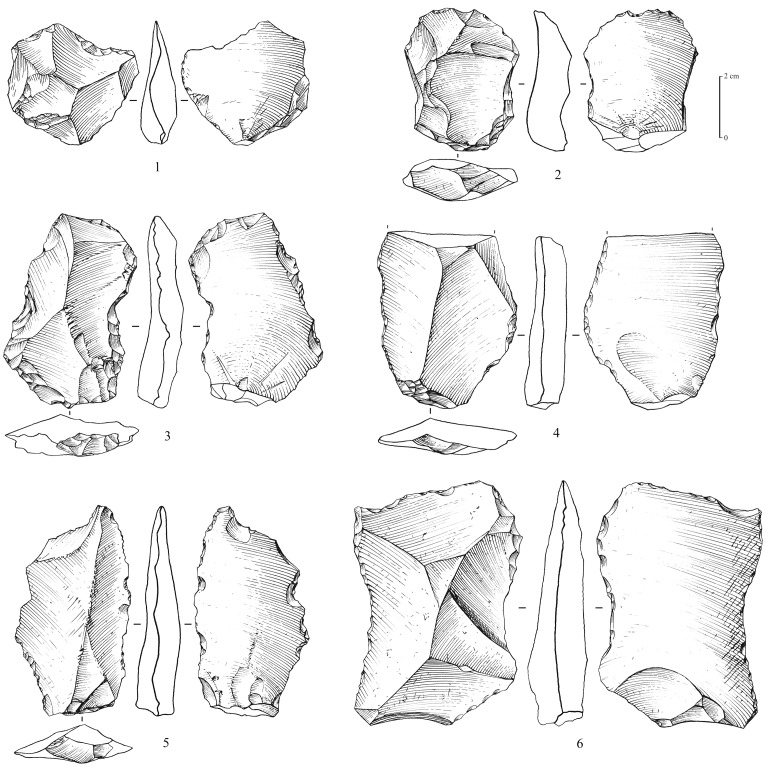
Levallois flakes from Mundafan, in chert. 1,2: debordant Levallois flake; 3–6: centripetal (preferential?) Levallois flakes. Drawings by G. Devilder, CNRS.

**Figure 8:**
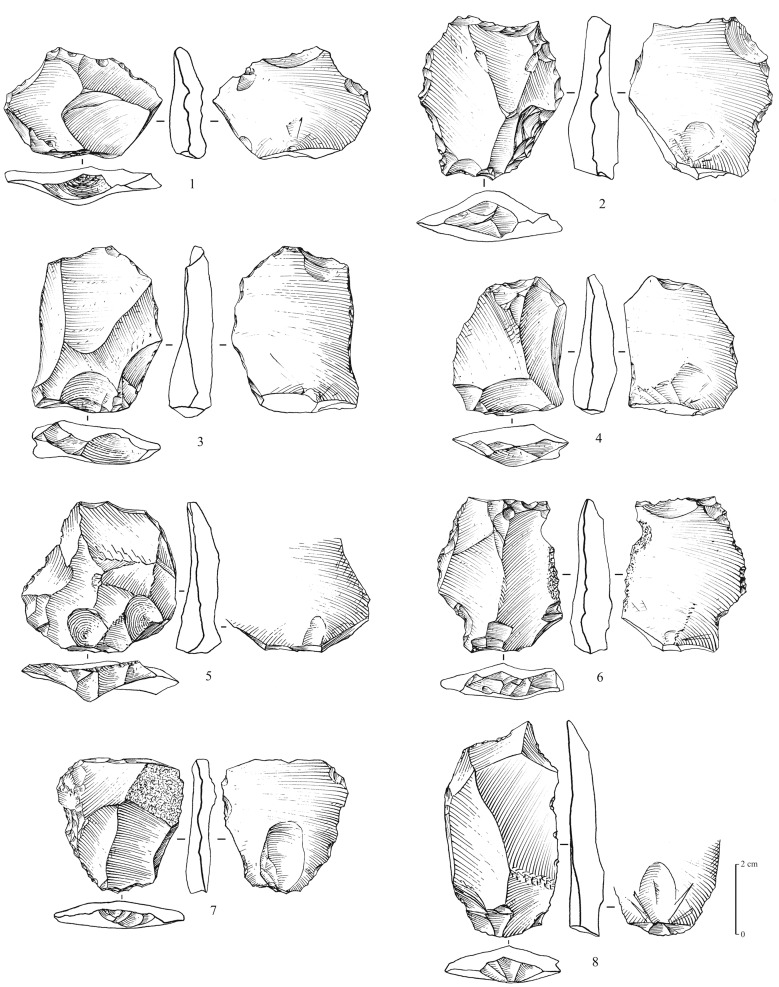
Levallois flakes from Mundafan, in chert. 1–8: centripetal (preferential?) Levallois flakes, 2 and 7 are retouched. Drawings by G. Devilder, CNRS.

**Figure 9:**
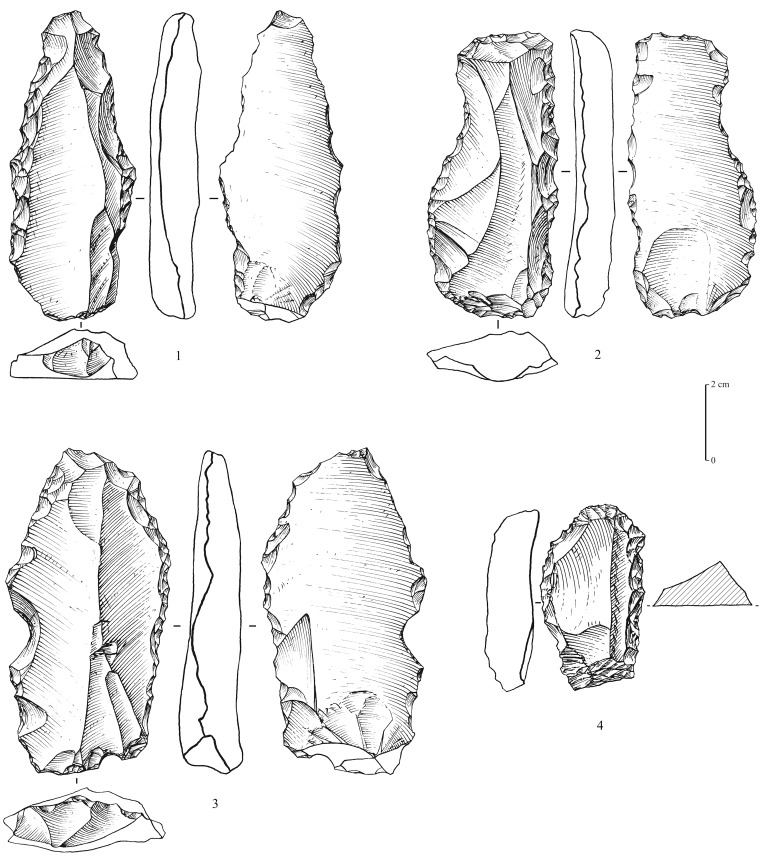
Middle Palaeolithic retouched tools from Mundafan, in chert. 1–4: retouched elongated thick flakes with facetted butts. Drawings by G. Devilder, CNRS.

Recent fieldwork led to the identification of 5 sites (surface scatters) yielding Middle Palaeolithic lithic assemblages (MDF-01, 02, 05, 13, 17; Fig. 3A). Non-diagnostic lithics of uncertain period were identified at 8 sites, most probably composed of mixed Middle Palaeolithic and Neolithic assemblages. This is the first time that technologically diagnostic Middle Palaeolithic assemblages have been reported and recognized at Mundafan. In comparison to Neolithic artifacts, the older Middle Palaeolithic artifacts may be distinguished by more rounded edges and arisses and greater surface patina. The technology is typical of the Arabian Middle Palaeolithic with the presence of the preferential Levallois reduction method with centripetal preparation, as well as the recurrent centripetal Levallois reduction method, as observed on 11 Levallois cores at MDF-01, MDF-10, MDF-17 (Fig. 6). The Mundafan prepared core technology is broadly similar to cores found in other Middle Palaeolithic sites in the Peninsula (e.g. JQ-1, JKF-1, JSM-1 sites at Jubbah: [21], [22]; AK-22 site at Al-Kharj: [75]; Hadramawt and Dhofar regions sites: [10], [76]. Fragmentary and whole Levallois flakes (n = 26, at MDF-01, 02, 05, 16, 17) were recovered (fig. 7 and 8), indicating the use of the Levallois single preferential flaking method, with centripetal preparation, and the Levallois recurrent method. Most of the Levallois blanks, probably produced by hard direct percussion, illustrate fine faceting in platform preparation. This is also visible on four debordant flakes at MDF-01. Core sizes are generally small (ranging from 50×37×10 to 65×62×19 cm), attesting of intensive debitage operations, as Levallois flakes could be much larger, showing a selection, and the availability, of bigger chert blocks by knappers. A few retouched tools on Levallois blanks were identified. They are generally characterized by a direct and denticulate retouch, as observed on some thick elongated flakes (Fig. 9). Their attribution to the Middle Palaeolithic is unsure, but most probable as the technology (dorsal scar patterns, facetted butts, retouch by direct hard hammer percussion, general dimensions) and the patina are suggestive of this period.

While some of the archaeological sites are found around the margins of the lake, many are found on the lake sediments, some towards the center of the basin. This indicates that the Middle Palaeolithic occupation of the region was not restricted to the periods of maximum humidity represented by lake high stands but shows that they were also present when the lake levels were low. To fully understand such observations considerably more work on both short term (i.e. seasonal) and longer term fluctuations of lake depth and area need to be conducted.

### Neolithic

**Figure 10:**
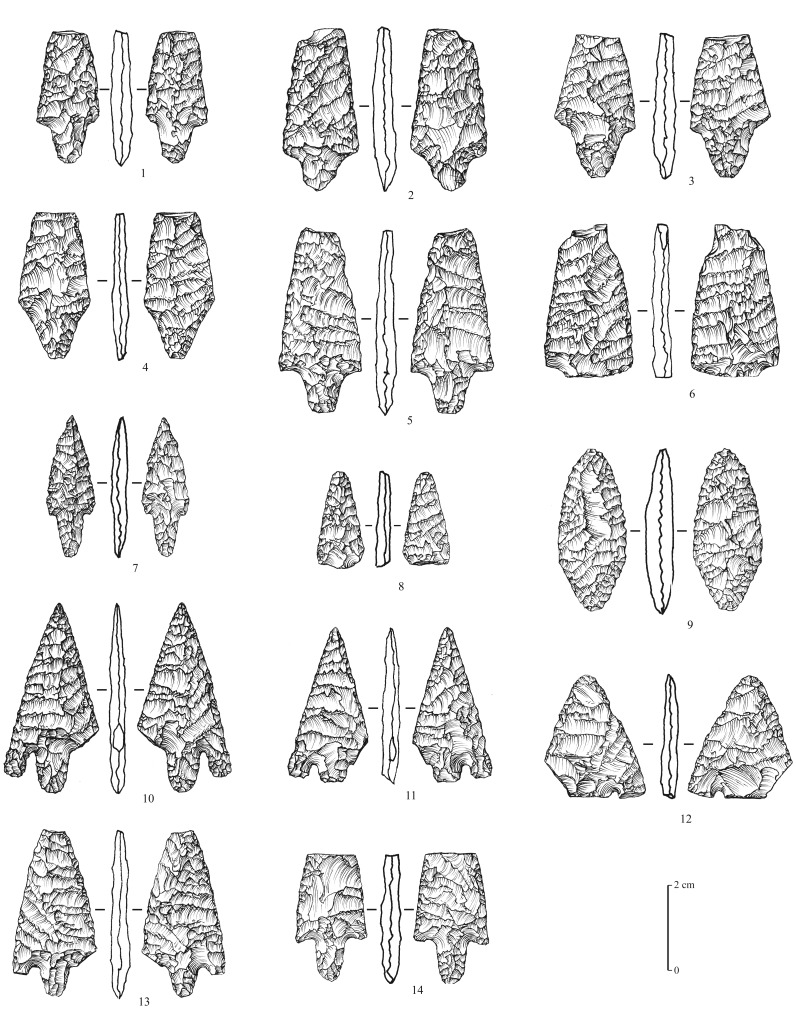
Neolithic arrowheads from Mundafan, in chert (sites MDF-12, 20, 21). 1–8: flat bifacial tanged projectile points with symmetrical section and shoulders, 9: flat bifacial piece (preform of a projectile point?), 10–14: flat bifacial tanged projectile points with symmetrical section and wings. Drawings by M. Leroyer, CNRS.

**Figure 11:**
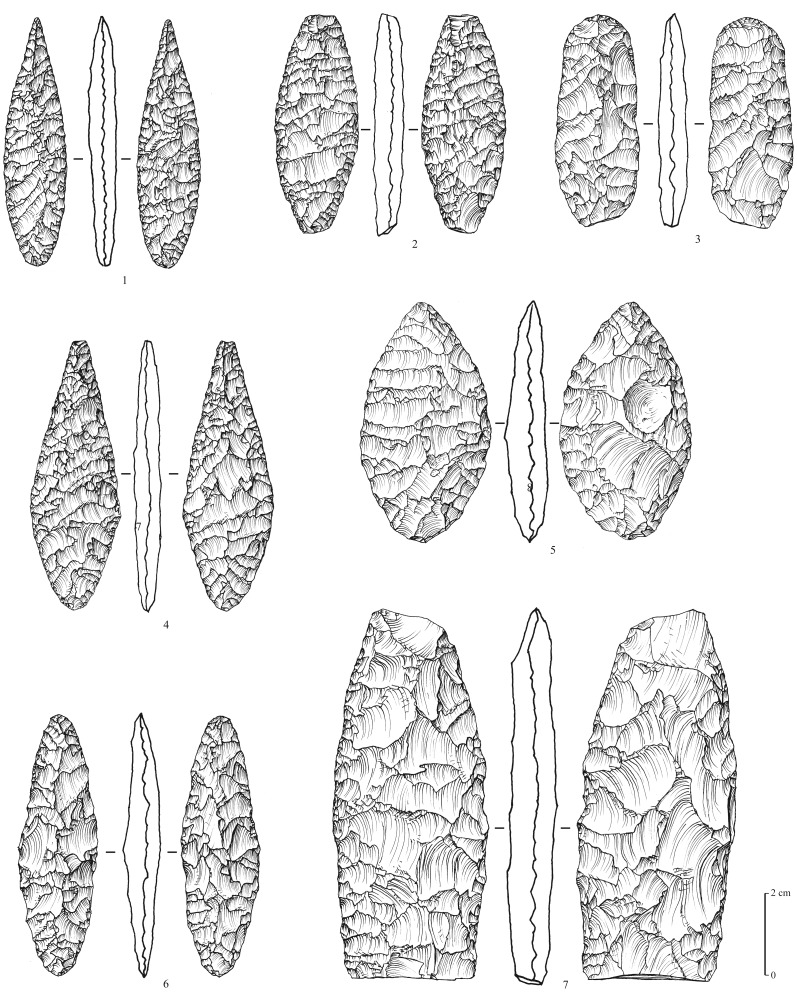
Neolithic bifacial foliates from Mundafan, in chert (sites MDF-16, 20, 21). **Drawings by M. Leroyer, CNRS.**

**Figure 12:**
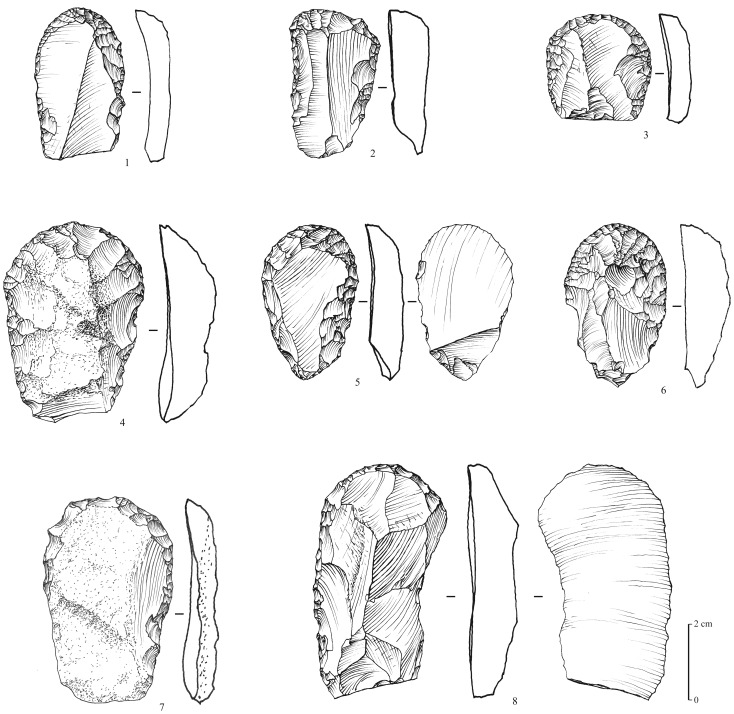
Neolithic thumbnail-shaped end-scrapers from Mundafan, in chert. **Drawings by M. Leroyer, CNRS.**

A total of 8 surface sites are attributed to the aceramic Neolithic of the Rub’ al-Khali tradition (or Desert Neolithic, [72], a techno-cultural facies that still needs to be clearly defined, but that is obviously different from other facieses known southwards of the desert in Oman and Yemen [11], [12]. The material collected, exclusively composed of lithic artefacts, is characterized by several types of tools, with a strong bifacial component. Arrowheads are numerous (n = 36, at MDF-16, 20, 21). They are shaped by the application of the pressure technique, with exquisite negative flake scar removals, creating a very thin symmetrical profile. The points consist of flat bifacial tanged arrowheads, with two main sub-types: with and without wings (Fig. 10). Small foliated bifaces (n = 18, at MDF-12, 20, 21), may have been used as ‘daggers’ and spear points (Fig. 11). End-scrapers and other typical Neolithic tools are similar to those found in numerous sites in Oman and Yemen. They are typically thumbnail-shaped, and consist of a single semi-circular active surface made by direct retouch, and sometimes by the pressure technique (Fig. 12). Other more ubiquitous types appear to be expediently made, and show irregular retouching.

**Figure 13:**
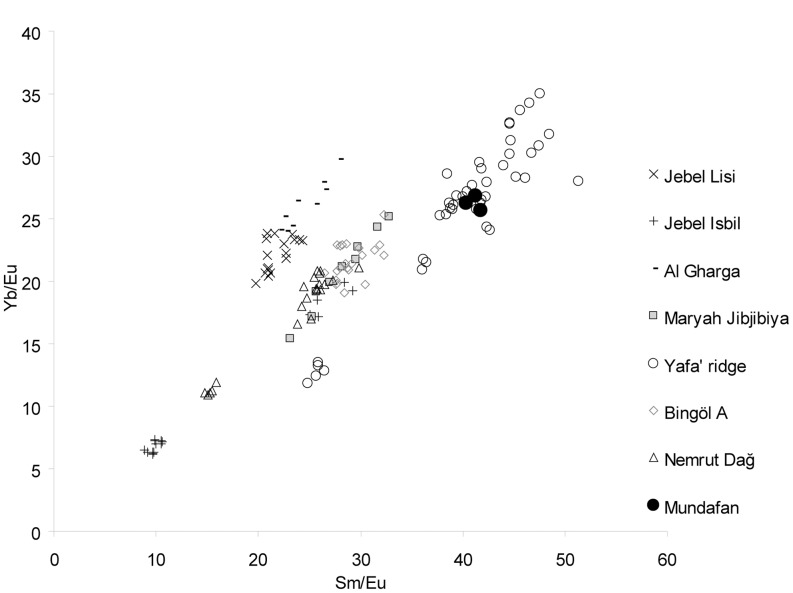
Obsidian analyses. Binary diagram Sm/Eu-Yb/Eu for the three analysed obsidian artifacts from Mundafan MDF-20 and for the main peralkaline obsidian sources from Anatolia and South Arabia.

During the survey, three obsidian flakes were collected at MDF-20, and although rare, such items were recognized to be potentially valuable for assessing site to source transport distances. The composition of the obsidian artifacts was examined using Laser Ablation High Resolution Inductively Coupled Plasma Mass Spectrometry (LA-HR-ICP-MS) (see for methods: [77]) at the CNRS/IRAMAT facilities in Orléans, France. Analytical results show that the three Mundafan samples are derived from a peralkaline obsidian source. The comparison of their composition with an obsidian source reference dataset from the Mediterranean, Anatolia, Trans Caucasia, South Arabia and East Africa, shows that the only peralkaline source that matches the composition of the three artefacts (by combining different element contents or ratios) is that of Yafa’ ridge in highland Yemen, Southwest Arabia [78] (Table 3 and Fig. 13). This source is ∼450–460 km in a straight line from Mundafan.

**Table 3:** Chemical compositions of the obsidian artefacts from Mundafan and average compositions of the geological obsidians from Bingöl, Nemrut Dağ and Yafa’ridge.

Oxides in % Element in ppm	Obsidian artefacts			Obsidian sources		
	Mundafan 1	Mundafan 2	Mundafan 3	Nemrut	Bingöl A	Yafa'ridge
Li	65	67	60	98	99	74
B	15	16	15	103	155	11
Na_2_O	4.7%	4.6%	4.4%	5.3%	5.2%	4.6%
MgO	0.0015%	0.0015%	0.0013%	0.0073%	0.0083%	0.0060%
Al_2_O_3_	13%	12%	13%	11%	12%	12%
SiO_2_	75%	75%	74%	69%	73%	75%
K_2_O	4.1%	4.0%	3.9%	3.9%	3.8%	4.4%
CaO	0.26%	0.26%	0.25%	0.35%	0.37%	0.24%
Sc	12	13	12	10	8	12
TiO_2_	0.16%	0.15%	0.16%	0.22%	0.19%	0.18%
Ti	940	908	959	1304	1146	1088
Mn	384	388	388	727	564	429
Fe_2_O_3_	3.2%	3.3%	3.2%	4.1%	3.6%	3.3%
Fe	22384	23028	22229	28388	25379	23422
Zn	214	240	200	206	186	185
Rb	251	254	242	235	223	249
Sr	0.20	0.19	0.17	0.57	0.80	0.48
Y	101	101	111	114	129	91
Zr	943	940	1038	1113	1198	926
Nb	113	117	119	71	56	113
Cs	4.1	4.3	4.0	9.2	14	3.8
Ba	0.46	0.57	0.39	3.4	2.8	3.3
La	74	67	82	92	91	74
Ce	167	160	178	187	188	176
Pr	18	17	19	19	20	17
Nd	72	69	80	78	83	74
Sm	18	17	19	17	19	17
Eu	0.43	0.43	0.47	0.63	0.65	0.44
Gd	17	16	18	17	19	17
Tb	3.0	2.9	3.3	3.1	3.5	2.7
Dy	19	18	21	20	22	18
Ho	3.8	3.8	4.3	4.1	4.8	3.5
Er	11	11	12	12	14	11
Tm	1.6	1.6	1.8	1.8	2.0	1.5
Yb	11	11	13	13	13	11
Lu	1.6	1.6	1.8	1.8	2.0	1.5
Hf	22	22	25	24	25	21
Ta	6.6	6.6	7.1	4.4	4.0	6.4
Th	28	28	32	29	32	26
U	6.8	7.2	7.0	11	12	6.3

Oxides concentrations are expressed in weight percents and elements concentrations in part per million.

Ostrich egg shells were also identified at MDF-13 and MDF-14. Ostrich fossils have not been recovered at Mundafan ([37], p.182). The ostrich adorns rock art in other Holocene settings such as at the Jubbah palaeolake [79], [80], with their extinction in Arabia occurring in the early 20^th^ century AD [81]. At least one fragment of a grinding stone was noted at MDF-07, perhaps suggesting grain milling in the Neolithic, or any other undated activity involving a material transformation (e.g. work of bones, shells, ochre, ore).

## Discussion and Conclusions

Survey in the Mundafan palaeolake basin revealed, for the first time, Middle Palaeolithic occupations. Recovery of Middle Palaeolithic assemblages corresponds with recent environmental and geoarchaeological studies that indicate at least three lacustrine wet phases in MIS 5 [23]. The main diagnostic lithic technology observed is the preferential Levallois reduction method, which is also present at the Jubbah palaeolake during MIS 5 (JQ-1, JSM-1 and JKF-1 sites: [21], [22]) and at the Jebel Faya rock shelter at the transition between MIS 6–5e (Assemblage C: [6]). This technology is absent in MIS 3 in southwest Yemen (SD1, SD2 and AS1 sites: [8], [9]). Earlier dating for preferential Levallois in Arabia, in MIS 7, is possible, but still insufficiently represented owing to small sample size at the Jubbah palaeolake (JQ-1: [22]). Other preferential Levallois methods have been observed in Dhofar, including in the Nubian Complex, dated to at least ca. 106 ka [7], in Hadramawt and the southern fringe of the Rub’ al-Khali, Oman [10], and in central Saudi Arabia at Al-Kharj [75]. Nubian Complex technology has not yet been identified at Mundafan. We associate the Levallois component in Mundafan with the wet pluvials of MIS 5, most probably during the wetter events of MIS 5e (ca. 125 ka), MIS 5c (ca. 100 ka) and MIS 5a (80 ka), when conditions were more favorable for hominin dispersals. The Middle Palaeolithic evidence thus provides empirical support for Rosenberg and colleagues assertion [23] that the dispersal of hominins into the Rub’ al-Khali occurred during ameliorated periods, and perhaps supports their claim for the expansion of *Homo sapiens* into this marginal environment.

The Mundafan Neolithic arrowheads are pertinent typological indicators, and although well known from surface sites [71], [72], no stratified sites have yet been reported. Closely comparable to the Mundafan examples, tanged arrowheads with wings have been found and dated to ca. 7,000–6,500 cal. BC at Khuzmum and the HDOR-561 site in Hadramawt [2], [12], [82], but more sites, both in south Arabia and from the Rub’ al-Khali need to be dated in order to figure out if this specific type was produced by one cultural group across a wide region. As a matter of fact, the tanged arrowheads with wings from Mundafan cannot be precisely dated with this one solely comparative occurrence. Absent at Mundafan are the typical south Arabian Neolithic arrowhead shapes, such as ‘trihedral points’ dating between 6,500–4,500 cal. BC [11], [83], and the ‘fluting technique’ dated to 6,000 to 5,500 cal. BC [12], [84]. In the absence of radiometric ages for the Neolithic sites in the Rub’ al-Khali at present, we attribute the Neolithic phase at Mundafan to ca. 8–6 ka BP, i.e. in the 7th-6th millennia cal. BC, or during the Holocene wet phase, broadly dating from 10.5 to 6 ka BP. The Mundafan Neolithic appears to represent a different cultural facies compared to the Neolithic site complexes known in Yemen, Oman and the UAE, where trihedral and fluted points are well known. Another possible explanation for the technological and stylistic differences might be the result of a later age for the Mundafan industries, perhaps corresponding to the late Neolithic, i.e. the 5th millennium BC, or to the last dated period for a wet phase at the lake [23]. Comparable sites in terms of geographical setting are found in Yemen central desert of Ramlat as-Sab’atayn (al-Hawa: [18], [19]) where Neolithic campsites have been discovered along palaeolake shores [85].

The Mundafan Neolithic sites do not appear to be sedentary locations on the basis of the absence of architectural features, grindstones, domesticated faunal remains, and relatively low artifact densities. The prevalence of projectiles and other weaponry is probable evidence of hunting activities. Mundafan would have been a favorable setting for short-term hunting along the lakeshore. The presence of rare obsidian artifacts demonstrates Mundafan’s participation in long-distance mobility systems that included relations with the obsidian-rich mountainous zones of Yemen, some 400–500 km away from the site.

The archaeology associated with Lake Mundafan is not an isolated occurrence (Fig. 4). Neolithic sites are found throughout much of the study area, providing evidence for extensive human occupation and associated humidity throughout the region. There is also evidence for a widespread Middle Palaeolithic and, to a lesser extent, possible Upper Palaeolithic occupation in the region, though the technologies associated to them do not have such an extensive distribution as the Neolithic, with most sites located in the headwaters of the major river system in the Asir and Tuwayq Mountains and their foothills, with a noticeable lack of sites in the Rub’ al-Khali. The broad spatial distribution of the archaeology and the evidence for past humidity suggests that there were many routes for hominin dispersal to and from the Mundafan region. The large river systems in the vicinity of the lake provide a number of attractive dispersal routes to and from the site. Access can be readily achieved primarily along rivers either from the Red Sea and over the Asir Mountains, from the Arabian Sea via the Wadi Hadramawt or the Arabian Gulf via the Wadi ad-Dawasir.

New environmental studies, remote sensing research and archaeological reconnaissance survey at Mundafan is beginning to shed light on the relationship between climate change and human presence. Currently, there is no clear evidence for the presence of Upper Palaeolithic or Late Palaeolithic industries at Mundafan, seemingly confirming an Arabia wide pattern [38], and suggesting that human groups were not able to survive at Mundafan during arid and hyper-arid periods, especially in MIS 4 and 2, and the ‘debated pluvial’ in MIS 3 [20]. New interdisciplinary investigations at Mundafan are planned for the near future, with the aim of identifying closer connections between environments and stratified archaeological sites in dateable contexts.
